# Integration of bioinformatics to biodegradation

**DOI:** 10.1186/1480-9222-16-8

**Published:** 2014-04-27

**Authors:** Pankaj Kumar Arora, Hanhong Bae

**Affiliations:** 1 School of Biotechnology, Yeungnam University, Gyeongsan 712-749, Republic of Korea

## Abstract

Bioinformatics and biodegradation are two primary scientific fields in applied microbiology and biotechnology. The present review describes development of various bioinformatics tools that may be applied in the field of biodegradation. Several databases, including the University of Minnesota Biocatalysis/Biodegradation database (UM-BBD), a database of biodegradative oxygenases (OxDBase), Biodegradation Network-Molecular Biology Database (Bionemo) MetaCyc, and BioCyc have been developed to enable access to information related to biochemistry and genetics of microbial degradation. In addition, several bioinformatics tools for predicting toxicity and biodegradation of chemicals have been developed. Furthermore, the whole genomes of several potential degrading bacteria have been sequenced and annotated using bioinformatics tools.

## Background

Millions of toxic chemicals have been produced for use in a variety of industries [1]. These chemicals have often been released into the environment due to anthropogenic activities, where they contaminate soil and water [2]. Furthermore, many chemicals persist in the environment, causing severe problems to living organisms; accordingly, it is crucial that these compounds be removed from the environment [2].

Biodegradation is the break-down of chemicals or xenobiotic compounds by microbes and plants [3]. Biodegrading microbes degrade toxic chemicals via either mineralization or co-metabolism [4]. In the process of mineralization, microbes completely degrade toxic chemicals by utilizing them as carbon and energy sources, whereas co-metabolism results in biotransformation of toxic compounds into less toxic compounds [4,5].

Microbial remediation is an emerging technology for the removal of toxic chemicals from the environment [4-6]. A large number of microbes capable of utilizing toxic chemicals as their sole sources of carbon and energy have been isolated, many of which break complex chemical compounds down to carbon dioxide and water through a series of chemical reactions catalyzed by microbial enzymes [5-8], such as monooxygenases, dioxygenases, reductases, deaminases, and dehalogenases. The genes encoding these enzymes have been identified in a variety of microbes and cloned into bacteria to increase the efficiency of bioremediation. The degradation of a specific toxic chemical requires a specific microbe that depends on the structure of that chemical and the presence of the enzyme systems in bacteria for degradation of the compound. Therefore, knowledge regarding chemicals (classification, identification, environmental properties, toxicity, distribution, and associated risks) as well as their microbial biodegradation (xenobiotics degrading bacteria, enzymes, genes, proteins) can improve bioremediation process.

Bioinformatics, which has been incorporated into each branch of life sciences, provides a platform for researchers to develop valuable computational tools for human and environmental welfare [9,10]. In the last few decades, bioinformatics has been integrated with biodegradation and several bioinformatics tools useful in the field of biodegradation have been developed. These include databases [11-14], chemical toxicity prediction systems [15,16], biodegradation pathway prediction systems [17-20], and next-generation sequencing [21-24]. Here, we discuss the relationship of bioinformatics tools with biodegradation.

### Databases

In recent years, an increasing number of databases have been developed to provide information regarding chemicals and their biodegradation. These databases may be characterized into two categories: chemical databases and biodegradative databases. Table 1 provides a list of various chemical databases that enable classification identification and risk assessment of chemicals or describe their environmental properties, toxicity and distribution.

**Table 1:** List of chemical databases

**Database**	**Description**	**Reference**
Databases for chemical identification, structure and classification		
ChemIDplus	Information about 370,000 chemicals.	[25]
ECHA Classification & Labeling Inventory	Information about the classification and labeling of substances reported and registered by manufacturers and importers.	[26]
NCLASS (the Nordic N-Class Database on Environmental Hazard Classification)	Information describing chemicals that have been or are currently being considered by the European commission on classification and labeling for environmental effects.	[27]
Databases describing environmental properties of chemicals and their toxicity, distribution, management and risk of occupational disease		
Hazardous Substances Data Bank (HSDB)	Toxicology information for 5,000 chemicals.	[28]
Toxicology Literature Online (TOXLINE)	References derived from toxicology literature.	[29]
Chemical Carcinogenesis Research Information System (CCRIS)	Carcinogenicity and mutagenicity tests for 8,000 chemicals.	[30]
Developmental and Reproductive Toxicology Database (DART)	References related to developmental and reproductive toxicology literature.	[31]
Genetic Toxicology Data Bank (GENE-TOX)	Data related to genetic toxicology for 3,000 chemicals.	[32]
Integrated Risk Information System (IRIS)	Data describing hazard identification and dose–response assessments of about 500 chemicals.	[33]
International Toxicity Estimates for Risk (ITER)	Risk information for 600 chemicals from authoritative groups worldwide.	[34]
TOXNET	A cluster of databases on toxicology, hazardous chemicals, environmental health, and toxic releases.	[35]
SuperToxic	A comprehensive database of about 60,000 toxic compounds.	[36]
Acutoxbase	This innovative database may be used for in vitro acute toxicity studies	[37]
Comparative Toxicogenomics Database (CTD)	This database describes genetic bases by which environmental chemicals affect human diseases.	[38]
Carcinogenic Potency Database	This database contains the results of 6540 chronic, long-term animal cancer tests on 1547 chemicals.	[39]
International Uniform Chemical Information Database (IUCLID)	Physico-chemical properties, environmental fate, toxicity and ecotoxicity of 2,600 chemicals.	[40]
Haz-Map	An occupational health database that provides information on chemicals and related occupational diseases.	[41]
TOXMAP	A Geographic Information System that provides the amount and location of toxic chemicals released into the environment using maps of the United States.	[42]
Toxics Release Inventory (TRI)	Data focused on specific toxic chemicals and their management as waste.	[43]
The Household Products Database	Information on the health effects of 13,000 consumer brands.	[44]
European chemical Substances Information System (ESIS)	Information about chemicals covering a variety of aspects.	[45]
ECOTOX (AQUIRE, PHYTOTOX, TERRETOX)	Chemical toxicity data for aquatic life, terrestrial plants and wildlife.	[46]
eChemPortal	Information on properties of chemicals including toxicity, ecotoxicity, environmental fate and behavior and physical chemical properties.	[47]
EnviChem	Environmental properties of chemicals.	[48]
Aggregated Computational Toxicology Resource (ACToR)	All publically available chemical toxicity data.	[49]
EPA Human Health Benchmarks for Pesticides (HHBP)	Information describing human health benchmarks for pesticides to determine whether the detection of a pesticide in drinking water or source waters for drinking water indicate potential health risks.	[50]
EPA Office of Pesticide Programs’ Aquatic Life Benchmarks (OPPALB)	Aquatic ecotoxicity benchmarks values from risk assessments developed by the EPA for individual pesticides.	[51]
Chemical Safety Information from Intergovernmental Organizations - INCHEM	Internationally peer reviewed information derived from intergovernmental organizations describing chemicals commonly used throughout the world	[52]
JECDB: Japan Existing Chemical Data Base	Toxicity test reports from Japan's existing chemicals safety program.	[53]
Substances in Preparations In the Nordic countries (SPIN)	Provides information regarding chemicals in the products of Nordic Countries	[54]
US EPA: Substance Registry Services (SRS)	A central system of the USEPA and the portal for discovering chemical information at the EPA	[55]

Biodegradative databases store information related to biodegradation of chemicals including xenobiotics-degrading bacteria, metabolic degradation pathways of toxic chemicals, enzymes and genes involved in the biodegradation. These databases include the University of Minnesota Biocatalysis/Biodegradation database (UM-BBD), a database of biodegradative oxygenases (OxDBase), Biodegradation Network-Molecular Biology database (Bionemo), MetaCyc, and BioCyc.

**Figure 1:**
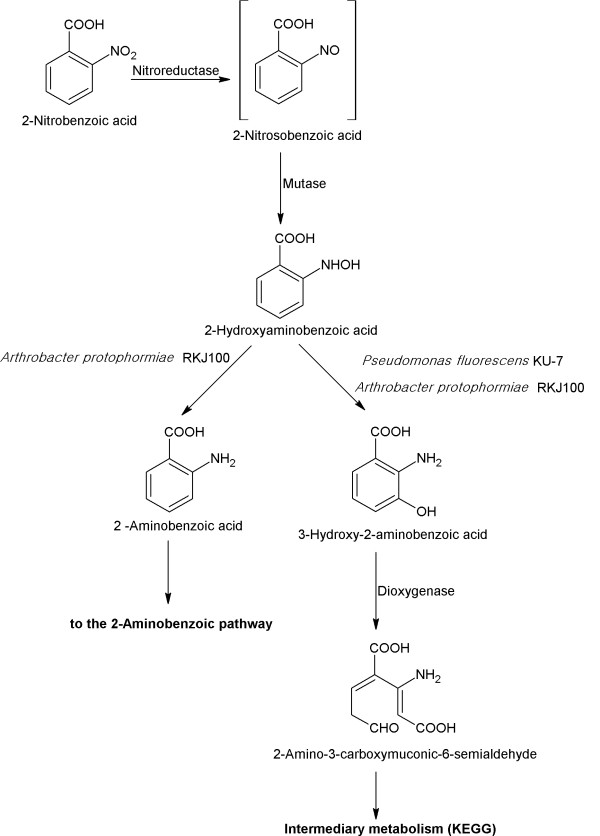
**2-Nitrobenzoic Acid Pathway Map at the UM-BBD (**http://umbbd.ethz.ch/onb/onb_map.html**).**

The UM-BBD is a well-known database in the field of biodegradation that is freely available at http://umbbd.ethz.ch/. This database provides information pertaining to multiple fields of interest including microbes, biotransformation rules, enzymes, genes and reactions involved in microbial degradation [11]. This database mainly focuses on the metabolic pathways of xenobiotic compounds which are available in text as well as graphic formats. Pathways represent multisteps enzymatic reactions in a series initiating from the starting compound and proceeds via the formation of intermediates. There is a diversity of the bacteria that can degrade a chemical compound via different pathways. All known pathways for a single compound are included in the UM-BBD metabolic pathway page (known as pathway map) of a particular compound with the information of the bacteria and enzymes involved in the degradation of that compound. Figure 1 represents the UM-BBD pathway map of 2-nitrobenzoic acid where two bacterial degradation pathways are present. Both pathways were initiated with the formation of 2-hydroxylaminobenzoic acid that further degraded via two different pathways in different bacteria. Currently, the UM-BBD database comprises (i) 219 microbial degradation pathways; (ii) 1503 chemical reactions; (iii) 993 enzymes; (iv) 543 microbes; (v) 250 biotransformation rules; (vi) 50 functional groups; (vii) 76 reactions of naphthalene 1, 2- dioxygenase and (viii) 109 reactions of toluene dioxygenase. This database is cross linked to several others including ExPASy, BRENDA, Enzyme and NCBI to provide information describing genes and enzymes involved in the degradation of xenobiotic compounds [11].

Another database, OxDBase (http://www.imtech.res.in/raghava/oxdbase/), which was developed by the CSIR-Institute of Microbial Technology, Chandigarh, India, stores information regarding oxygenases derived from published literature and databases [12]. Oxygenases are the most important enzymes involved in aerobic degradation of aromatic compounds [12]. There are two types of oxygenases, monooxygenases and dioxygenases. Monooxygenases catalyze incorporation of one atom of molecular oxygen into substrate whereas dioxygenases catalyze incorporation of two atoms of molecular oxygen [12]. Dioxygenases are further divided into aromatic ring hydroxylating dioxygenases (ARHD) and aromatic ring cleavage dioxygenases (ARCD). ARHD catalyze hydroxylation of aromatic rings, whereas ARCD catalyze ring cleavage of aromatic rings [12]. ARCDs are further divided into extradiol and intradiol. Intradiol ARCDs cleave aromatic rings between two hydroxyl groups, whereas extradiol cleaves rings between hydroxylated carbons and adjacent non-hydroxylated carbons [12]. OxDBase provides information about 237 distinct oxygenases, including monooxygenases (118) and dioxygenases (ARCD, ARHD, intradiol and extradiol) (119). All enzyme entries contain information about (a) reaction(s) in which enzymes are involved, (b) their common names and synonyms, (c) structures and gene links, (d) families and subfamilies, (e) literature citations and (f) links to several external databases including the Kyoto Encyclopedia of Genes and Genomes (KEGG, http://www.genome.jp/kegg/), UM-BBD, BRENDA, and ENZYME. This database is user-friendly and increases our understanding of aerobic degradation of aromatic compounds [12].

The Bionemo database (http://bionemo.bioinfo.cnio.es) was developed by the structural Computational Biology Group at the Spanish National Cancer Research Center [13]. Bionemo is a manually curated database that provides information regarding proteins and genes involved in biodegradation metabolism [13]. The protein information involves sequences, domains and structures for proteins, whereas the genomic information involves sequences, regulatory elements and transcription units for genes [13]. Bionemo complements UM-BBD, which focuses on the biochemical aspects of biodegradation [13]. Bionemo has been developed by manually associating sequence database entries to biodegradation reactions based on the information extracted from published articles [13]. Information related to the transcription units and their regulation of biodegradation genes is linked to the underlying biochemical network. This database is composed of (i) 145 biochemical pathways, (ii) 945 reactions in which 342 reactions are with associated complexes, (iii) 537 enzymatic complexes, (iv) 1107 proteins, (v) 234 microbial species (vi), 212 transcription units (vii), 90 transcription factors, (viii) 90 effectors, (XI) 128 TF DNA binding sites and (X) 100 promoters. Like other databases, Bionemo is cross linked to the following databases: (i) UMBBD for metabolic reaction; (ii) GenBank for DNA sequences; (iii) Uniport for protein; (iv) NCBI Taxonomy for microbial species and (v) PubMed for references [13]. The information provided by Bionemo may be helpful for cloning, primer design and directed evolution experiments. The full database is downloadable as a PostgresSQL dump [13].

MetaCyc is a database of metabolic pathways derived from the scientific experimental literature that comprises more than 2097 experimentally determined metabolic pathways from more than 2460 different organisms. This is the largest curated database of metabolic pathways of all domains of life [14]. This database provides information regarding the metabolic pathways involved in primary and secondary metabolism with associated compounds, enzymes and genes [14]. This database is freely available at http://metacyc.org/. MetaCyc can be used for multiple scientific applications. Specifically, it can (i) provide reference data for computational prediction of the metabolic pathways of organisms from their sequenced genomes, (ii) support metabolic engineering, (iii) facilitate comparison of biochemical networks, and (iv) serve as an encyclopedia of metabolism [14]. This database was developed and curated by the BioCyc group at SRI international.

BioCyc (http://biocyc.org/) is a collection of more than 2988 organism-specific Pathway/Genome Databases (PGDBs). Each PGDB contains the full genome and predicted metabolic pathway of a single organism [14]. The pathway tool software predicts pathways using MetaCyc as a reference database [14]. The predicted metabolic pathway contains information about metabolites, enzymes, and reactions. In addition, BioCyc PGDBs contain information about predicted operons, transport systems and pathway-hole fillers [14]. BioCyc pathway tool based web sites offer multiple tools for querying and analysis of PGDBs, including analysis of gene expression, metabolomics, and other large-scale datasets [14]. This database was developed by the Bioinformatics Research Group at SRI International.

### Pathway prediction systems

Only a small portion of toxic chemicals have been tested for their microbial degradation; however, a large number of toxic chemicals remain unexplored for biodegradation testing, despite the fact that they have been released into the environment. Knowledge regarding the degradation of these compounds is essential to determination of the fate of these chemicals in the environment. In such cases, computational tools may be used to predict biodegradation pathways for these toxic chemicals. Several pathway prediction systems have been developed using either non-biochemically based or biochemically based methods [56,57]. Non-biochemically based pathway prediction systems use statistical inference methods to generate reactions between compounds [57]. These systems include machine learning methods [58], the Bayesian method [59], comparative genomics [60] and metabolic network alignment [61]. These methods are very useful to identify missing links in the network [57,62]. The disadvantage of these methods is that these reactions are based on statistical inference alone; therefore; many of them could be biochemically infeasible [57]. Biochemically-based pathway prediction systems work on knowledge based biotransformation rules. Table 2 summarizes the role of various pathway prediction systems useful in the field of biodegradation. Here, we are presenting some details of biochemically based pathway prediction systems.

**Table 2:** Pathway prediction systems

**System**	**Comments**	**Reference**
UM-PPS	Predicts microbial degradation pathways for xenobiotic compounds based on biotransformation rules.	[17]
PathPred	Predicts pathways for microbial biodegradation of environmental compounds and biosynthesis of plant secondary metabolites.	[18]
Biochemical Network Integrated Computational Explorer (BNICE)	Predicts novel thermodynamic feasible pathways on the basis of reaction rules of the enzyme commission classification system.	[19,63]
DESHARKY	A Monte Carlo algorithm that identifies metabolic pathways from target compounds using a database of known enzymatic reactions. Also provides amino acid sequences of corresponding enzymes from phylogenetically closely related organisms.	[64]
From Metabolite to Metabolite (FMM)	Online tool that predicts the pathway between two compounds based on the KEGG database.	[21]
CarbonSearch	Algorithm that identifies pathways within existing metabolic networks by tracking the conservation of atoms moving through them.	[65]
OptStrain	Computational framework that advises on optimization of the host’s metabolic network to add a particular metabolic pathway by adding or deleting reactions	[66]
Metabolic Tinker	Predicts all paths between two compounds	[21]

The UM-BBD-Pathway Prediction System (PPS) is a part of UM-BBD that may be accessed at http://umbbd.ethz.ch/predict/. The PPS can be used to predict metabolic pathways for microbial degradation of chemical compounds [17]. Predictions are based on biotransformation rules derived from reactions found in the UM-BBD database or in the scientific literature [17]. Users can predict both aerobic and anaerobic degradation pathways of chemicals and can select whether they will view all or only the more likely aerobic transformations [17]. Users can also obtain the most accurate prediction for those compounds similar to compounds with biodegradation pathways that have been reported in the scientific literature [17]. For example, the degradation pathways of 4-nitrophenol have been thoroughly investigated, while those of 2-fluro-4-nitrophenol and 2-bromo-4-nitrophenol have not. However, the structures of 2-fluro-4-nitrophenol and 2-bromo-4-nitrophenol are similar to 4-nitophenol; therefore, PPS can provide very accurate predictions for degradation of 2-flouro-4-nitrophenol and 2-bromo-4-nitrophenol. For the prediction, users may enter a compound into the system by either drawing the structure and generating SMILES or entering SMILES directly.

Another pathway prediction system, PathPred (http://www.genome.jp/tools/pathpred/), is a knowledge based prediction system that uses data derived from the Kyoto Encyclopedia of Genes and Genomes (KEGG) in the form of the KEGG REACTION database and KEGG repair database [18]. The KEGG REACTION database contains not only all known enzymatic reactions taken from the IUBMB enzyme nomenclature, but also additional reactions taken from the KEGG metabolic pathways [18]. KEGG RPAIR is a collection of biochemical structure transformation patterns (RDM patterns) for substrate–product pairs (reactant pairs) in KEGG REACTION. PathPred is a web-based server that predicts plausible enzyme-catalyzed reaction pathways from a query compound using information regarding RDM patterns and chemical structure alignments of substrate-product pairs. This server provides plausible reactions and transformed compounds and displays all predicted reaction pathways in a tree-shaped graph. PathPred based predictions are very accurate for compounds that have biochemical similarity to KEGG compounds [18]. PathPred contains reference pathways (i) for microbial biodegradation of environmental compounds and (ii) for biosynthesis of plant secondary metabolites. The users can select one of the reference pathways according to their purpose [18]. There are multiple user friendly methods for searching a pathway for query. Specifically, a query compound can be input (i) in the MDL mol file format, (ii) the SMILES representation, or (iii) by the KEGG compound identifier. In the case of the xenobiotics biodegradation reference pathway, users should use the compound to undergo biodegradation as a query, while in the case of the reference pathway of biosynthesis of secondary metabolites the query should be the end product of biosynthesis. The prediction results are linked to genomic information [18]. The PathPred server provides new and alternative reactions, regardless of whether enzymes for these reactions are known or not. If the enzyme is unknown, users can use the E-zyme tool (http://www.genome.jp/tools/e-zyme/) to assign a possible EC number (up to the EC sub-subclass). After assigning EC numbers, it is also possible to search the putative genes in the genome based on sequence similarity of known genes with the same EC sub-subclass [18].

Biochemical Network Integrated Computational Explorer (BNICE) is computational approach for development of novel pathways based on the reaction rules of the Enzyme Commission classification system [19]. BNICE generates all possible pathways from a given target or starting molecule. In the next step, BNICE screens out all possible pathways for thermodynamic feasibility based on the Gibbs free energies of the reaction and selects feasible novel thermodynamic pathways [57]. Soh and Hatzimanikatis [57] suggested that the pathways generated by BNICE can be further evaluated using established pathway analysis approaches, such as thermodynamics-based flux balance analysis (FBA) GrowMatch, which allows investigation of the overall effects of these novel pathways on metabolic network performance in host organisms [57]. FBA can help predict maximum yield, phenotypic changes, effects of gene knockouts, changes in bioenergetics of the system for metabolic engineering, synthetic biology, and biodegradation of xenobiotics [57]. BNICE can be applied in multiple areas: (i) to discover novel pathways for metabolic engineering; (ii) for ‘retrosynthesis’ of metabolic chemicals, (iii) to investigate evolution between metabolic pathways of various organisms; (iv) to analyze metabolic pathways; (v) for mining of omics data; (vi) to select targets for enzyme engineering; and for (viii) analysis of degradation pathways of xenobiotic compounds [57].

**Figure 2:**
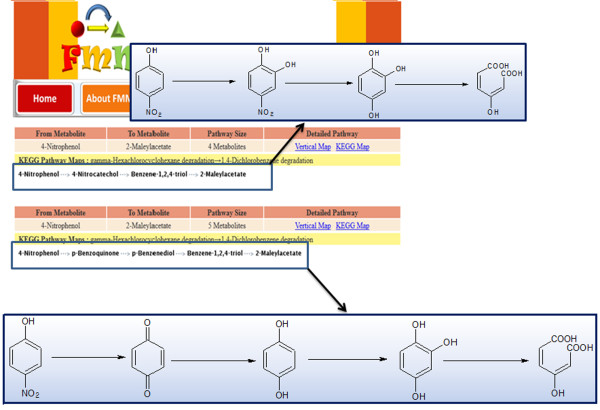
**All pathways between 4-nitrophenol and 2-maleylacetate using FMM webserver.** FMM generates two pathways between 4-nitrophenol and 2-maleylactate. One pathway via formation of 4-nitrocatechol and other via p-benzoquinone.

From Metabolite to Metabolite (FMM) is a web server freely available at http://FMM.mbc.nctu.edu.tw/ that is able to search all possible pathways between known input and output compounds among various species based on the KEGG database and other integrated biological databases [20]. FMM can generate combined pathway maps by combining the KEGG maps and KEGG LIGAND information [20]. This server provides information regarding the corresponding enzymes, genes and organisms and provides a platform called “comparative analysis,” in which metabolic pathways can be compared between several species. FMM is an efficient tool for drug production, biofuel production, synthetic biology and metabolic engineering [20]. For biodegradation purposes, we can search metabolic pathways of only those xenobiotic compounds for which information is available in the KEGG database. One example is presented in Figure 2, which shows the search of a pathway between 4-nitrophenol and 2-maleylacetate.

A recently developed web tool, Metabolic Tinker (http://osslab.ex.ac.uk/tinker.aspx) can be used to design synthetic metabolic pathways between user-defined target and source compounds [21]. Metabolic Tinker uses a tailored heuristic search strategy to search for thermodynamically feasible paths in the entire known metabolic universe [21]. The program contains a directed graph known as Universal Reaction Network (URN), which represents the entire set of known reactions and compounds from the Rhea database [21]. Nodes and edges on this graph represent metabolites and reactions, respectively, and thus the entire graph represents the current known metabolic universe [21]. Metabolic tinker searches possible biochemical paths between two compounds within this URN using standard search algorithms developed in computer science and graph theory [21]. The Rhea/CHEBI identification codes of both the source and target compounds are needed to complete the search [21].

### Computational methods for predicting chemical toxicity

The computational methods for estimating chemical toxicity are evolving rapidly [67]. In recent years, several models have been developed in which computational programs have been used to predict the toxicity of chemical compounds [22-24,67,68]. Quantitative structure-regulatory activity relationship (QSAR) models calculate toxicity based on the physical characteristics of the structure of chemicals such as the molecular weight or the number of benzene rings (molecular descriptors) using mathematical algorithms [69]. Following are the some examples of commercial and publicly-available models:

•*Sarah Nexus* for prediction of the mutagenicity of chemicals [70].

•*VirtualToxLab* for prediction of the toxic potential (endocrine and metabolic disruption, some aspects of carcinogenicity and cardiotoxicity) of drugs, chemicals and natural products [71].

•*Toxicity Estimation Software Tool* (TEST) for prediction of the acute toxicity of organic chemicals based on their molecular structures [72].

•*TOPKAT* for prediction of the ecotoxicity, mutagenicity, and reproductive/developmental toxicity of chemicals [73].

•*Ecological Structure Activity Relationships* (ECOSAR) for estimation of the aquatic toxicity (acute short-term), toxicity and chronic (long-term or delayed) toxicity of industrial chemicals to aquatic organisms such as fish, aquatic invertebrates, green algae and aquatic plants by using computerized structure activity relationships [74]

•*Estimation Programs Interface* (EPI) suite for prediction of physical/chemical properties and environmental fate (eco-toxicity). The software calculates chemical property data using programs including KOWWIN, AOPWIN, HENRYWIN, MPBPWIN, BIOWIN, KOCWIN, WSKOWWIN, WATERNT, BCFBAF, HYDROWIN and ECOSAR [75].

•*CAESAR* for assessment of chemical toxicity under the REACH [76].

•*ToxiPred:* A server for prediction of aqueous toxicity of small chemical molecules in *Tetrahymena pyriformis*[77].

### Genome sequences of xenobiotic degrading bacteria

The automated Sanger method for sequencing is known as first generation sequencing, whereas newer methods developed for sequencing are considered next generation sequencing (NGS) [78]. Commercially available NGS technologies include Roche/454, Illumina/Solexa, SOLiD/Life/APG, Helicos BioSciences, and the Polonator Instrument [78].

The initial steps of NGS involve generation of short reads and their subsequent alignment to a reference genome. The latter step is crucial for NGS technologies, and a variety of computational tools have been applied for genome sequence assembly including SSAKE [79], SOAPdenovo [80], AbySS [81], and Velvet [82]. Once the sequence reads are assembled into contigs, the next steps are gene prediction and functional annotation. The most common gene prediction system for microbial systems is GLIMMER (Gene Locator and Interpolated Markov ModelER), which identifies the coding region on the microbial genome based on interpolated Markov models [83,84]. The predicted coding region sequences may be analyzed and evaluated manually or by automatic annotation software to identify the homologous genes. A variety of automatic pipelines are available for bacterial annotation, including online tools such as RAST [85], BASys [86], WeGAS [87] and MaGe/Microscope [88], as well as offline tools such as AGeS [89], DIYA [90] and PIPA [91]. Furthermore, MICheck [92] may be used to check for syntactic errors in annotated sequences.

NGS ignited a revolution in biodegradation and bioremediation with the concept of “from genomics to metabolomics.” Bacterial genomics is the study of the whole genomes of bacteria in which genes involved in biodegradation and other metabolic processes can be predicted. The whole genomes of several xenobiotic degrading bacteria have been sequenced using NGS technology, and several xenobiotic-degrading genes have been identified through gene predictions and annotation of the bacterial genomes [93-97]. *In silico* analysis of the bacterial genome leads to prediction of metabolic pathways for the biodegradation of xenobiotics and gives a holistic view of the metabolic network of particular bacteria [98]. Several metabolic pathways may be predicted from the genomes of xenobiotic degrading bacteria [99,100]. For example, the whole genome of C*upriavidus necator* JMP134 (previously known as *Ralstonia eutropha*, Strain JMP134), which utilizes a variety of aromatic and chloroaromatic compounds as the sole carbon and energy sources, was sequenced and several genes coding the enzymes involved in the degradation of various xenobiotic compounds were identified [100,101]. The genome of strain JMP134 comprises four replicons (two chromosomes and two plasmids) with a total of 6631 protein coding genes. The *C. necator* JMP134 genome contains 300 genes putatively involved in central ring-cleavage pathways of various aromatic compounds [101].

In *silico* analysis of the genome of *Pseudomonas putida* KT2440 showed that the presence of the following pathways for degradation of aromatic compounds: (i) the *ortho* pathway for the catabolism of protocatechuate (*pca* genes) and catechol (*cat* genes), (ii) the phenylacetate pathway (*pha* genes), and (iii) the homogentisate pathway (*hmg* genes) [102]. Additionally, the gene clusters for catabolism of N-heterocyclic aromatic compounds (*nic* cluster) and in a central *meta*-cleavage pathway (*pcm* genes) were also identified in the genome of this microorganism [102].

Whole-genome sequences are not only useful for prediction of genes and their functions, but also for identification of novel biocatalysts [98]. Combining the genomic approach with proteomic approaches will lead to new insights into metabolism at the organism level [98]. Kim et al. [103] used metabolic, genomic and proteomic approaches to construct a complete and integrated pathway for pyrene degradation in *Mycobacterium vanbaalenii* PYR-1 and identified 27 enzymes that were used to construct a complete pathway for pyrene degradation based on genomic and proteomic data [103].

## Conclusion

Several databases have been developed for providing the information on chemicals and their biodegradation. Users can use these databases to retrieve the information according to their research interests. For example, users can retrieve the information on toxicity, risk assessment, and environmental properties of the chemicals using chemical databases. Furthermore several bioinformatics tools have been developed for the prediction of the toxicity of chemicals. Users can use these tools for prediction of the toxicity of the chemicals. In addition, several pathway prediction systems are available for predicting the degradation pathways for those chemicals whose degradation pathways are not known in literature. The UM-BBD and PathPred are well known pathway prediction systems for biodegradation purpose. Using these pathway prediction systems, users can predict not only the degradation pathways, but also identify enzymes involved in the degradation pathways. This approach would be very useful for metabolic engineering and also to develop the strategy for bioremediation. The major problem related to the pathway predictions is that the predicted pathways are yet not experimentally verified. In the future, experimental studies should be carried out to verify the predicted pathways. Furthermore, the genomes of the several xenobiotics-degrading bacteria have been sequenced using NGS and the genes and enzymes involved in the biodegradation have been identified using gene-annotation. In future, molecular techniques along with bioinformatics tools may provide new insights into the genetics of the biodegradation.

## Competing interests

The authors declare that they have no competing interests.

## Authors’ contributions

PKA collected all the relevant publications, arranged the general structure of the review, drafted the text and produced figures. HHB revised and formatted the review and also help to draft the manuscript. All authors read and approved the final manuscript.
